# Seroprevalence and risk factors of porcine cysticercosis: A cross-sectional study in Indonesia

**DOI:** 10.14202/vetworld.2022.30-34

**Published:** 2022-01-17

**Authors:** Annytha Detha, Putri Pandarangga, Yunita Nope

**Affiliations:** 1 Laboratory of Veterinary Disease and Veterinary Public Health, Faculty of Veterinary Medicine, Nusa Cendana University, Jl. Adi Sucipto, Penfui, PO BOX 104, Kupang 85001, East Nusa Tenggara, Indonesia; 2 Laboratory of Pathology, Faculty Veterinary Medicine, Nusa Cendana University, Jl. Adi Sucipto, Penfui, PO BOX 104, Kupang 85001, East Nusa Tenggara, Indonesia; 3 Livestock Service Office of Kupang City, Provincial Government, East Nusa Tenggara, Indonesia.

**Keywords:** cysticercosis, epidemiology, risk factor, seroprevalence

## Abstract

**Background and Aim:**

Cysticercosis is a zoonotic disease with a global concern. Estimation of the prevalence and identification of potential risk factors are necessary for the prevention and control of the disease. This study aimed to estimate the seroprevalence of cysticercosis and the correlation of the increased prevalence with several potential risk factors.

**Materials and Methods:**

The seroprevalence of cysticercosis was conducted using an enzyme-linked immunosorbent assay (ELISA), developed by the Institute of Tropical Medicine Antwerp, to detect *Cysticercus cellulosae*. This study used serum samples from 62 pigs taken from two regencies on Timor Island. The data analysis was performed using SPSS software 20.0 (IBM Corp., NY, USA) to evaluate ELISA results and the strength of the relationship between risk factors and the prevalence of disease using the odds ratio (OR).

**Results:**

Serum samples from 18 out of the 62 pigs were found to be positive; the seroprevalence of cysticercosis was 29%. The results showed that an extensive farming system led to a higher prevalence of cysticercosis compared to an intensive farming system, namely, 10 out of 18 (56.6%), and that the possibility of identifying cysticercosis in pigs in an extensive farming system was 5 times greater than that in pigs in an intensive farming system. In addition, the results showed that nine out of 18 households who did not have toilet facilities were found to be seropositive, indicating a significant relationship between the risk factor of toilet availability with cysticercosis in pigs, with an OR of 4.5. In addition, the results showed that there was no significant relationship between the risk factor of the feed source and the prevalence of cysticercosis in pigs.

**Conclusion:**

It can be concluded that the seroprevalence of cysticercosis was 29% in domestic pigs of Timor Island. The risk factors of an extensive pig farming system andtoilet availabilityin community houses were significantly related to the possibility of cysticercosis on Timor Island.

## Introduction

Cysticercosis is a disease caused by the larvae of *Taenia solium*, which is a dangerous zoonotic parasite [1,2] that has adverse economic impacts on the local community [3]. Cysticercosis has adverse effects on human health, including epilepsy as the main symptom, nerve system disorders, and hydrocephalus [4,5]. However, cysticercosis in pigs usually has non-specific subclinical symptoms [6,7] and clinical symptoms [8]. It also causes direct adverse effects, such as farmers’ health, and indirect losses, such as loss of production time [4].

Despite its high prevalence in developing regions, cysticercosis is not well studied [9-12]. The disease is endemic in several regions, such as Africa [13], Latin America [14], America [15], Asia [16,17], and in various nations in Southeast Asia, including Thailand, Singapore, the Philippines, Laos, Myanmar [18], and Indonesia [6,16,19-22]. Cases of cysticercosis in Indonesia have been reported in several regions, such as Papua, Bali, Sumatra, Sulawesi, Java Island, Kalimantan, and Flores [18]. In Indonesia, cysticercosis is reported to be found in both humans and animals [6,23]. Research carried out in Bali, using an indirect enzyme-linked immunosorbent assay (ELISA) method, revealed a case of *Cysticercus cellulosae* in local pigs that were slaughtered at a slaughterhouse in Denpasar City. Antibodies to *C. cellulosae* were detected in 33 out of 270 serum samples of the slaughtered pigs [24].

The prevalence of cysticercosis is inextricably linked to several risk factors that drive the transmission of cysticercosis. Some of the risk factors related to the prevalence of cysticercosis that have been reported are the farming system [25], the level of public sanitation [26], unhygienic feed given to livestock [27], and a large pig population [28]. Indeed, the pig population on Timor Island increases annually, while the people on the island still use a traditional, extensive pig farming system. In addition, farmers generally pay little attention to the health of livestock and the cleanliness of livestock housing, and most farmers still feed household leftovers to pigs. These conditions are considered risk factors for the prevalence of cysticercosis on Timor Island. Nonetheless, until now, there has been no information available regarding the prevalence of cysticercosis in this region.

This study aimed to estimate the seroprevalence of cysticercosis and the relationship between the prevalence of cysticercosis in pigs and the risk factors that drive the spread of this zoonotic disease.

## Materials and Methods

### Ethical approval and Informed consent

The study was approved by Ethics Commission for the Use of Animals in Research and Education, Faculty of Veterinary Medicine, Udayana University (approval no. 242/KE-PH/V/2016). Verbal consent was obtained from each participant before the study.

### Study period and location

This cross-sectional study was conducted from January to December 2017. Pig blood sample was collected from the pig farm in Timor Island, Timor Tengah Selatan Regency, Timor Tengah Utara regency. The samples were processed at the Veterinary Laboratory of Animal Husbandry Department, Nusa Tenggara Timur (NTT) province.

### Study design

Cross-sectional study was conducted to estimate the seroprevalence of cysticercosis and the correlation of the increased prevalence of cysticercosis with potential risk factors including farming system, toilet availability, and feed source as described by Thrusfield and Brown [29]. The seroprevalence of cysticercosis was detected using an ELISA method. The study was divided into three stages: (a) Pig blood collection, (b) assessment of risk factors with questionnaires, and (c) conducting the ELISA assays and data analysis.

In the first stage, the pig blood serum was collected. The sample size was determined using the formula 4PQ/L^2^ with a 95% confidence level. The sample for this study consisted of serum from 62 pigs obtained from two regencies on Timor Island, namely, East South Central Regency and North Central Timor Regency. All serum samples were analyzed using the ELISA method to detect the existence of antigens of cysticercus.

The second stage was the assessment of risk factors using questionnaires. A total of 62 respondents have filled out the questionnaire. Respondents were selected based on the ownership of pigs that were sampled from the research. The answers helped us to determine the risk factors that were correlated with the prevalence of cysticercosis. The risk factors that were identified were the sex of the hog and the pig farming system. The third stage was the detection of cysticercus in hog blood sera using a direct sandwich ELISA (Bio-X Diagnostics, Institute of Tropical Medicine, Antwerp, Belgium), performed in the Veterinary Laboratory of Animal Husbandry Department, Nusa Tenggara Timur (NTT) province. This stage was used for the data analysis and to map the positive zone of cysticercosis in NTT.

This method used monoclonal antibodies to detect the antigens of *C. cellulosae* in the serum samples. A serum was positive for antigens of *C. cellulosae* if the ratio between the average absorbance value and the cutoff was ≥1000. The serum was negative for antigens of *C. cellulosae* if the ratio between the average absorbance value and cutoff was ≤0.300.

### Risk factor data collection

Risk factor data were obtained by assessing questionnaires distributed to respondents. The data were confirmed through direct observations of the condition of the pig housing and the sanitation conditions of the surrounding environment. The data obtained from the questionnaire included the type of pig farming system used, the type of feed given to the pigs, and the sanitation conditions of the farmers’ environment in terms of toilet availability.

### Statistical analysis

This study used a cross-sectional design with the data analyzed using SPSS software 20.0 (IBM Corp., NY, USA). The data obtained from the ELISA and the questionnaires were then tested in terms of correlation using the Chi-square test to determine p values and the strength of the relationship between risk factors and the prevalence of disease using the odds ratio (OR).

## Results and Discussion

The results of ELISA showed that 18 out of the 62 samples of pig blood serum were found positive for cysticercosis (29%). In addition, other regions in Indonesia, such as Bali [23], Papua, and North Sumatera [19,21], also reported a high seroprevalence of cysticercosis. Indeed, the prevalence of cysticercosis is likely to be found in most regions with a pig population, including Timor Island.

### Relationship between the risk factor of farming system and cysticercosis

The increasing prevalence of cysticercosis results from several risk factors. This research showed a higher prevalence of cysticercosis in extensive farming systems than with intensive farming systems, 10 out of 18 systems (56.6%). One of the risk factors for cysticercosis is extensive livestock farming, where hygiene practices are generally poor. The term “extensive rearing” means that farmers do not control the movement of livestock or control the types of feed consumed, which may be contaminated with *T. solium* larvae. The relationship between the risk factor of the farming system and the prevalence of cysticercosis in pigs is shown in Table-1.

**Table 1:** ELISA results for cysticercosis seroprevalence and risk factors.

Risk factors	ELISA		Chi-square	Odds ratio	Confidence interval

	Positive (%)	Negative (%)			
Farming system					
Extensive	10 (55.6%)	8 (18.2%)	0.03	5.625	1.686-18.763
Intensive	8 (44.4%)	36 (81.8%)			
Toilet availability					
Not available	9 (50.0%)	8 (18.2%)	0.01	4.5	1.355-14.944
Available	9 (50.0%)	36 (81.8%)			
Feed source					
Household Leftover food	14 (77.8%)	28 (63.6%)	0.28	2.000	0.562-7.119
Mixed feed	4 (22.2%)	16 (36.4%)			

There was a significant relationship (p<0.05) between the risk factor of the farming system and the prevalence of cysticercosis in pigs on Timor Island. The OR value was >1, meaning that the farming system was a risk factor that is likely to increase the prevalence of cysticercosis in pigs. The OR value of 5.625 indicates that the prevalence of cysticercosis in pigs that were bred in an extensive farming system was 5 times higher than that of pigs that were bred in an intensive farming system, with an associated confidence interval of 1.686-18.763.

The results of this study are consistent with research showing that the prevalence of cysticercosis in Africa was related to extensive farming system, such as a free range pig farming system [29]. The study also found that the traditional farming system may result in feeding with poor hygiene, resulting in high livestock mortality, low productivity, and poor reproduction. In addition, a study found that an extensive farming system without worming increased the prevalence of cysticercosis, which is linked to lower weight gain compared with pigs bred in an intensive farming system [30]. Another study has also confirmed that extensive rearing systems, which allow animals to roam freely during the day and scavenge, can create opportunities for infection through the consumption of *Taenia* contaminated feed or water [31]. This fact should be considered in eradicating the prevalence of cysticercosis and taeniasis.

### Relationship between risk factor of toilet availability and cysticercosis

This study showed that nine out of 18 households with no toilet available were positive for cysticercosis. Meanwhile, nine out of 36 households with toilet facilities available were positive for cysticercosis (Table-1). p<0.05 indicated a significant relationship between the risk factor of toilet availability and the prevalence of cysticercosis in pigs. The OR was 4.5. Therefore, it can be seen that the pigs bred by farmers who did not have toilet facilities in their household were 4.5 times more likely to be positive for cysticercosis than those bred by farmers who had toilet facilities, with confidence interval of 1.355-14.944.

This study’s results are in line with those from a study in Teso, Kenya, which showed that the risk factor of toilet availability in each household served as the determinant of cysticercosis [32]. Other studies also showed that households with no toilet available had a higher possibility of cysticercosis in pigs, 2.03 times higher than those with a toilet available [33,34]. Hence, the risk factor of toilet availability increases the prevalence of cysticercosis [25].

Risk factors related to toilet availability and the occurrence of cysticercosis in China caused pigs to have access to human waste; hence, poor livestock management and limited sanitation were risk factors associated with the occurrence of *T. solium* infections [35]. Similarly, a study conducted in Yunnan, China, which found a relationship between the availability of household toilets and cysticercosis, showed that toilet use could be a form of intervention to reduce cysticercosis [36]. Another study in China [27] also showed that absence of a toilet resulted in a greater chance of increasing cysticercosis; therefore, it is important to consider toilet availability as a factor in the intervention of the prevalence of this disease.

A study conducted in Nigeria [36] found that risk factors were open defecation and improper use of toilets; free range system to raise pigs; indiscriminate or unregulated slaughter; inadequate hygiene and inspection of meat; and consumption of undercooked pork for increasing the prevalence of cysticercosis. In addition, another study in Nigeria [37] also reported that the risk factor of not having toilet facilities resulted in a 4-fold increase in transmitting *T. solium*. These facts further emphasized the importance of toilet availability in significantly reducing the prevalence of cysticercosis.

### Relationship between risk factor of feed source and cysticercosis

This study employed the ELISA method showed that serum from 14 out of 42 pigs from households who fed leftover food to pigs were positive for cysticercosis. In contrast, the results of ELISA were positive to only four out of 20 pig serums from households who did not feed food leftovers to pigs. The relationship between the risk factor of feed source and the prevalence of cysticercosis in pigs is shown in Table-1.

There was no significant (p>0.05) relationship between the risk factor of the feed source and the prevalence of cysticercosis in pigs. However, this result is different from the results of research showing that animal feed sourced from household leftovers collected by farmers from surrounding communities was a risk factor for cysticercosis in livestock. There was a possibility of the feed being contaminated with *T. solium* larvae [37]. Another study also mentioned that animal feed was associated with cysticercosis [38], because feed sourced from household leftovers, such as uncooked tubers and vegetables, is likely to be contaminated with larvae because the community maintains the practice of defecating outdoors.

## Conclusion

This study showed that the seroprevalence of cysticercosis in domestic pigs on Timor Island was 29%. The risk factors such as extensive pig farming system and toilet availability were significantly associated with the possibility of cysticercosis on Timor Island.

## Authors’ Contributions

AD and PP: Designed the study, contributed to the analysis and interpretation of data, and wrote the manuscript. YN: Drafted and revised the manuscript. All authors have read and approved the final manuscript.
